# Enhanced osseointegration through direct energy deposition porous coating for cementless orthopedic implant fixation

**DOI:** 10.1038/s41598-021-01739-9

**Published:** 2021-11-16

**Authors:** Dong Jin Ryu, Ara Jung, Hun Yeong Ban, Tae Yang Kwak, Eun Joo Shin, Bomi Gweon, Dohyung Lim, Joon Ho Wang

**Affiliations:** 1 Department of Orthopedic Surgery, Inha University Hospital, 27 Inhang-Ro, Jung-Gu, Incheon, 22332 South Korea; 2 Department of Mechanical Engineering, Sejong University, 209 Neungdong-ro, Gwangjin-Gu, Seoul, 05006 South Korea; 3 Samsung Biomedical Research Institute, Samsung Medical Center, Sungkyunkwan University School of Medicine, Seoul, 06351 South Korea; 4 Department of Orthopedic Surgery, Samsung Medical Center, Sungkyunkwan University School of Medicine, 81 Irwon-Ro, Gangnam-Gu, Seoul, 06351 South Korea; 5 Department of Health Sciences and Technology, SAIHST, Sungkyunkwan University, Seoul, 06351 South Korea; 6 Department of Medical Device Management and Research, SAIHST, Sungkyunkwan University, Seoul, 06351 South Korea

**Keywords:** Biological techniques, Biotechnology, Medical research, Materials science

## Abstract

Direct energy deposition (DED) is a newly developed 3D metal printing technique that can be utilized on a porous surface coating of joint implants, however there is still a lack of studies on what advantages DED has over conventional techniques. We conducted a systematic mechanical and biological comparative study of porous coatings prepared using the DED method and other commercially available technologies including titanium plasma spray (TPS), and powder bed fusion (PBF). DED showed higher porosity surface (48.54%) than TPS (21.4%) and PBF (35.91%) with comparable fatigue cycle. At initial cell adhesion, cells on DED and PBF surface appeared to spread well with distinct actin stress fibers through immunofluorescence study. It means that the osteoblasts bind more strongly to the DED and PBF surface. Also, DED surface showed higher cell proliferation (1.27 times higher than TPS and PBF) and osteoblast cell activity (1.28 times higher than PBF) for 2 weeks culture in vitro test. In addition, DED surface showed better bone to implant contact and new bone formation than TPS in in vivo study. DED surface also showed consistently good osseointegration performance throughout the early and late period of osseointegration. Collectively, these results show that the DED coating method is an innovative technology that can be utilized to make cementless joint implants.

## Introduction

Orthopedic implants are medical devices that have long been used to repair fractures and defects in bones to restore their function. Among the various orthopedic surgeries that employ implants, total joint arthroplasty (TJA) is one of the most effective treatments for end-stage joint degeneration, and is hence widely applied to hip and knee joints^1^. Traditionally for TJA, implant fixation relies mostly on the cement method, which generally produces good clinical outcomes. However, the benefit of initial firm fixation can be diminished by persistent shear and tensile forces on the cemented area, which can eventually result in micromotions and loosening of the implanted components^2,3^. In addition, cement particles that are generated at the interface between the cement and implant can induce local inflammation^4^. The occurrence rate of late implant failure due to these complications has been very low, but a recent marked increase in life expectancy has led to a significant increase in the number of joint failures worldwide.

Consequently, there has been an increase in interest in the cementless biological fixation method. The cementless fixation method, which relies primarily on biological bone healing is already widely used for hip joints, and its application is gradually extending to knee joints^5^. As demand for clinical application is increasing, a lot of effort has been devoted to developing a technology that can integrate a highly functional porous structure on an implant surface^6–8^. In particular, porous coating methods are known to enhance osseointegration and improve mechanical fixation by creating a mechanical interlock via bone ingrowth into the porous structure. Therefore, multiple porous coating methods, including fiber bead sintering, diffusion bonding, and titanium plasma spray (TPS) have been developed and studied for more efficient bone healing^6,9,10^. However, these methods have some technical limitations. For example, porous layers porous layers prepared using the bead sintering and TPS methods are not thick enough for intact bone ingrowth, and are easily peeled off from the implant surface^11,12^. Recently, the powder bed fusion (PBF) method using 3D printing has been developed and been used for surface coating of an orthopedic implant. Although many studies have reported that PBF showed better than solid implants, there has been no study comparing osseointegration ability with other surface coating methods^8,13,14^.

Direct energy deposition (DED) is a 3D metal printing technique that can be utilized to integrate porous structures similar to that of human cancellous bone on a metal surface while maintaining its mechanical strength^9,15,16^. Another advantage of the DED technique is that two different materials can be incorporated by spraying a metal powder onto a base made from different types of metals^9,15^. Using two different materials, an implant that exploits the advantages of both materials, such as biocompatibility, osseointegration capability, and mechanical strength, can be designed^17^. For example, implants comprising a porous titanium (Ti) coating on a cobalt-chromium (CoCr) alloy base can be physiologically beneficial because Ti coatings have better biocompatibility than CoCr coatings, and the CoCr base is likely to produce less wear debris than a Ti or Ti-6Al-4 V alloy base^18–21^.

In this study, we conducted a systematic comparative study of porous coatings made using the DED, TPS, and PBF methods. Since the three coating methods (DED, TPS, and PBF) have fundamentally different coating principles, it is impossible to achieve similar or identical pore morphologies using these three coating methods. With this in mind, we intended to compare the functional differences that arise due to the characteristics of each coating, especially the unique pore morphology produced by each coating method. As the TPS and PBF methods are commercially available coating techniques that are already being applied to patients, through this study, we determined the effectiveness of DED coatings and their suitability for use in orthopedic implants. The scope of our study included the (1) evaluation of fatigue life, (2) verification of biocompatibility, (3) investigation of cellular adhesion properties in vitro, and (4) determination of osseointegration performance in vivo for each surface-coated implant.

## Results and discussion

### Characteristics of the porous structure

**Figure 1:**
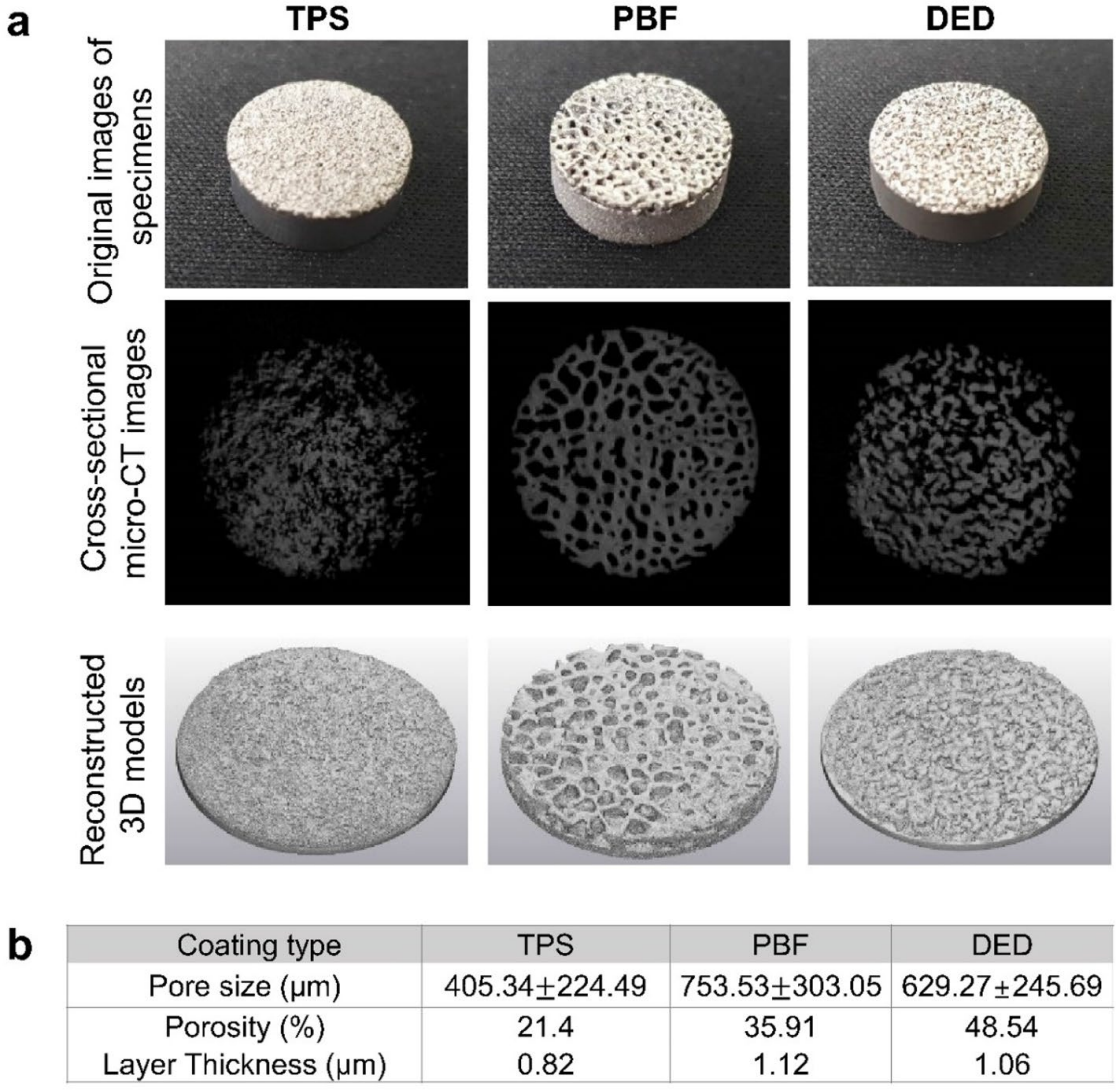
(**a**) Images of specimens (TPS, PBF, and DED): original images, cross-sectional images, and reconstructed 3D models. (**b**) Pore size, porosity, and thickness of coating layers.

To evaluate and compare structural characteristics in terms of the benefit of bone ingrowth, we analyzed the pore structure of three types of coatings (TPS, PBF, and DED) using micro-CT images (Fig. 1a). As shown in the cross-sectional images (second row of Fig. 1), the shape of the pores varied depending on the coating type. Overall, the pore structures of the PBF and DED specimens were larger than those in the TPS specimen (Fig. 1b). In addition, the porous layers of the PBF and DED specimens were thicker than the TPS layer. The pores had rounded and closed shapes with distinctive boundaries in the PBF specimen, whereas the pores in the TPS and DED specimens were somewhat connected to each other. Therefore, the PBF specimen appeared to have the morphological characteristics most similar to those of cancellous bone. However, the DED specimen had the highest average porosity (120% and 40% higher than those of the TPS and PBF specimens, respectively).

Both the porosity and pore size are known to play critical roles in bone ingrowth for cementless fixation. In particular, a high porosity and large pore size have been shown to promote bone in growth^11,22^. Therefore, the high average pore size and porosity of the DED coating can be advantageous for bone tissues to grow into the pore spaces.

### Fatigue cycle

We then determined how the fatigue life changed depending on the coating methods. Generally, materials with higher porosity tends to have lower fatigue life^23,24^. However, in our study, it is difficult to clearly identify how this porosity affected the fatigue life in each coating method because there are many other factors that can affect the fatigue life, such as surface roughness, density, and microstructure. It should also be taken into account that the structure or material of each specimen is different depending on the coating methods. In particular, the PBF specimen is entirely made of Ti-6Al-4V alloy, whereas TPS and DED specimens are made by coating pure Ti on the CoCr substrate. These differences should be taken into consideration.

**Figure 2:**
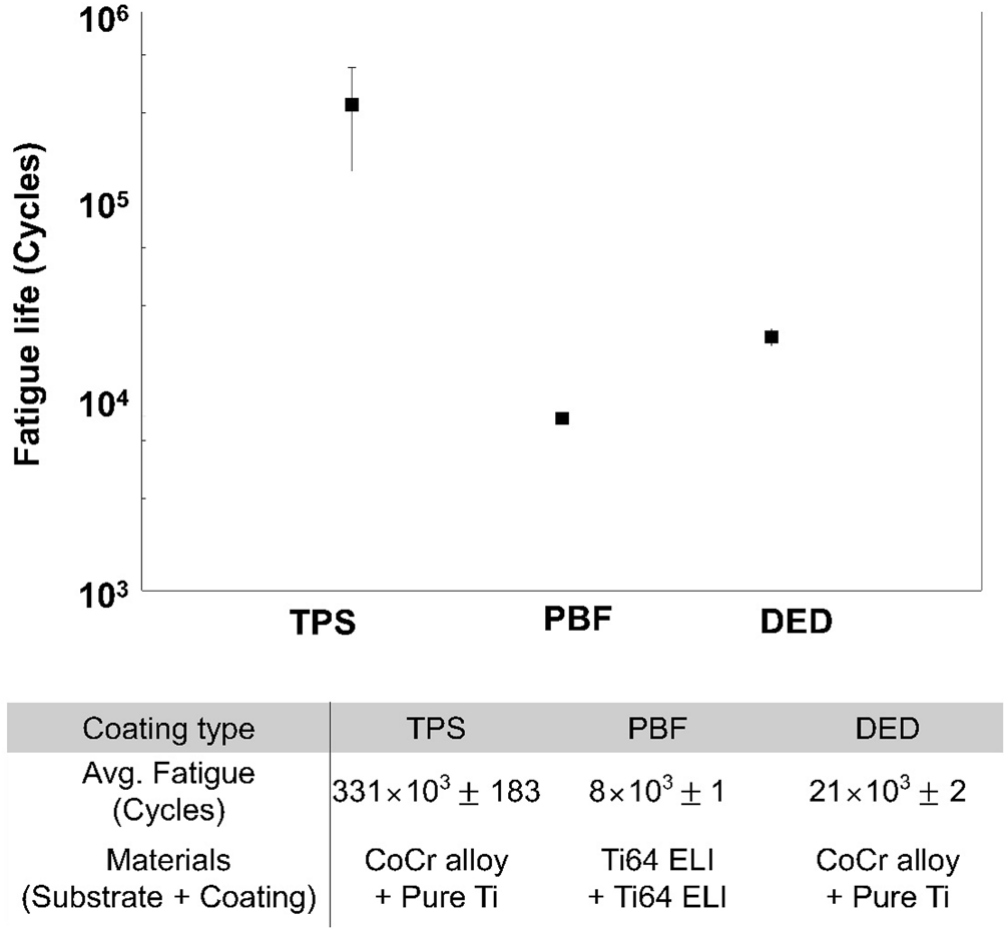
Fatigue life of TPS, PBF, and DED specimens.

The average fatigue life of the DED, PBF, and TPS specimens was 21 × 10^3^ ± 2 cycles, 8 × 10^3^ ± 1 cycles, and 331 × 10^3^ ± 183 cycles, respectively (Fig. 2). The TPS specimen had the longest, the DED specimen an intermediate, and the PBF specimen the shortest fatigue cycle of the three porous coated specimens. In this study, the PBF specimens were used as built. Therefore, it appears that high surface roughness and subsurface defects, which are unique characteristics of 3D printed materials, remain in the specimen, reducing the fatigue cycle of the PBF^25–27^. These subsurface defects can be eliminated by hot isostatic pressing (HIP) process and further enhance fatigue cycle^27^. In the case of TPS and DED, the CoCr alloy base parts were fabricated using the traditional machining method, but the fatigue cycle of the TPS specimen was approximately 15 times higher than that of the DED specimen. We assumed that this difference manifested during the porous coating process. Throughout the DED coating process, a high-power laser beam generates a melt pool across the surface of the base metal, and Ti metallic powder is partially melted and fused with a CoCr substrate^28,29^. During this process, due to the fast heating/cooling and the steep temperature gradients at the melt pool area, (1) intermetallic CoCr-Ti compounds can be formed, and (2) residual stress can be accumulated at the CoCr-Ti interface^30–34^. Since the intermetallic compounds are inherently brittle, these intermetallic compounds can reduce fatigue life^28,35^. In addition, the residual stress can accelerate the fatigue process, reducing the fatigue life of DED specimens^35^.

To investigate the structural alteration that can be induced by external loads at the CoCr-Ti interface, we imaged the cut cross-section of the specimens after the fatigue test (Supplementary Fig. S1). This cross-sectional study was not conducted on the PBF specimen because it comprised a single material (Ti-6Al-4V). In the TPS specimen, defects and debonding failure were observed at the CoCr/Ti coating interface (marked with red arrowheads in Supplementary Fig. S1). In the DED specimen, we did not observe the debonding failure but observed crack starting from the CoCr/Ti interface (this is marked with a blue arrowhead in Supplementary Fig. S1). Indeed, these cross-sectional images demonstrate the characteristics of DED coating that we mentioned above; the DED coating seems to allow strong metallurgical bonding between two different materials thanks to the heat-induced melt pool but appears to simultaneously increase the brittleness due to the intermetallic compounds at the interface^28^. Nevertheless, the HIP process can reduce intermetallic compounds in the CoCr-Ti interface and improve the fatigue cycle of DED coatings; therefore, we believe that DED can produce coatings of sufficient fatigue strength for clinical application^28,29^.

### Cell proliferation and ALP activity on porous coatings

**Figure 3:**
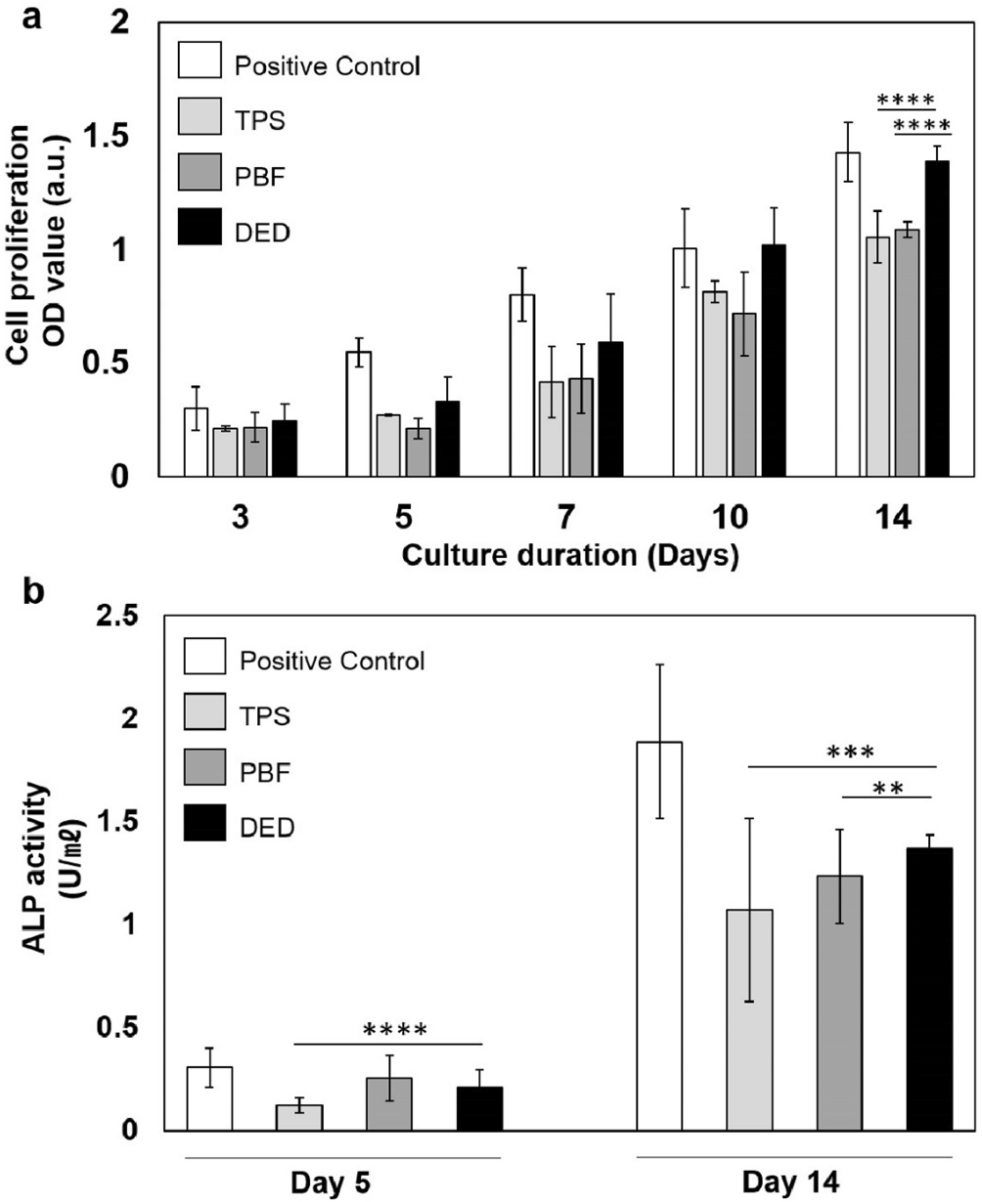
Cell proliferation and ALP activity of Saos-2 on different porous structures. Saos-2 cells were seeded at a density of 2.4 × 10^4^ cells/well and cultured for 14 days. (**a**) Cell proliferation was measured using CCK-8. (**b**) The enzyme activity of ALP within cells was measured on days 5 and 14. The trend of ALP activity was similar to that of cell proliferation (mean ± SD).

As mentioned above, high porosity in DED coatings seems to be beneficial for bone ingrowth; nevertheless, to further investigate how the geometry of pores can affect cell growth, we compared cellular proliferation on three different porous coatings (TPS, PBF, and DED) and a plastic surface (positive control) at various time points (3, 5, 7, 10, and 14 days after seeding). cell-counting-kit-8 (CCK-8) assay was performed, and cell numbers were measured using the optical density (OD) of CCK-8 reagent. As shown in Fig. 3a, the cell population increased in all the groups over time. However, the growth trend varied depending on the experimental conditions. The positive control exhibited the highest growth rate across all time points. The positive control was used to monitor the condition of the cells^36–38^. Therefore, for positive control samples, cells in a general cell culture plate, which is optimized for cell culture by increasing the hydrophilicity, whereas TPS, PBF, and DED specimens were not specially functionalized to facilitate cell attachment. Therefore, it seems to be natural that the positive control exhibited better proliferation than TPS, PBF, and DED. At the early time point (day 3), the TPS, PBF, and DED specimens showed similar cell numbers. From day 5, the cell number on the DED specimen started to exceed that on the TPS and PBF specimens, and, over time, even approached that on the positive control. On day 14, the OD value of the DED specimen was much higher than that of the TPS and PBF specimens, i.e., the cell population on the DED coating was much higher than that on the TPS and PBF coatings (Fig. 3a).

Then, we evaluated the level of osteoblast maturation in all the test groups (TPS, PBF, DED, and positive control) on days 5 and 14 post-seeding, by measuring the alkaline phosphatase (ALP) activity. The TPS specimen showed the lowest ALP activity, and the cells on the PBF and DED specimens showed similar levels of ALP activity on day 5. On day 14 post-seeding, the cells in the DED group exhibited significantly higher ALP activity than those in the TPS (*p* = 0.0075) and PBF (*p* = 0.023) groups (Fig. 3b).

To investigate whether the difference in cellular proliferation and maturation occurred due to the variation in the adhesion of cells to each porous coating type, we measured the number of cells attached to each porous coating in the early stage of culture. Interestingly, the cells seemed to adhere more to the TPS surface than to the PBF and DED surfaces within 4 h of seeding (Supplementary Fig. S2). According to a previous report, initial cell adhesion can decrease as the pore size increases^39^. Considering that the pore sizes of the DED and PBF coatings were greater than those of the TPS coating, the adherence of fewer cells to the DED and PBF surfaces seems reasonable. This result also suggests that the superior cell growth rate on DED coatings is not due to their high cell adhesion performance.

### Cell morphology on porous coatings

**Figure 4:**
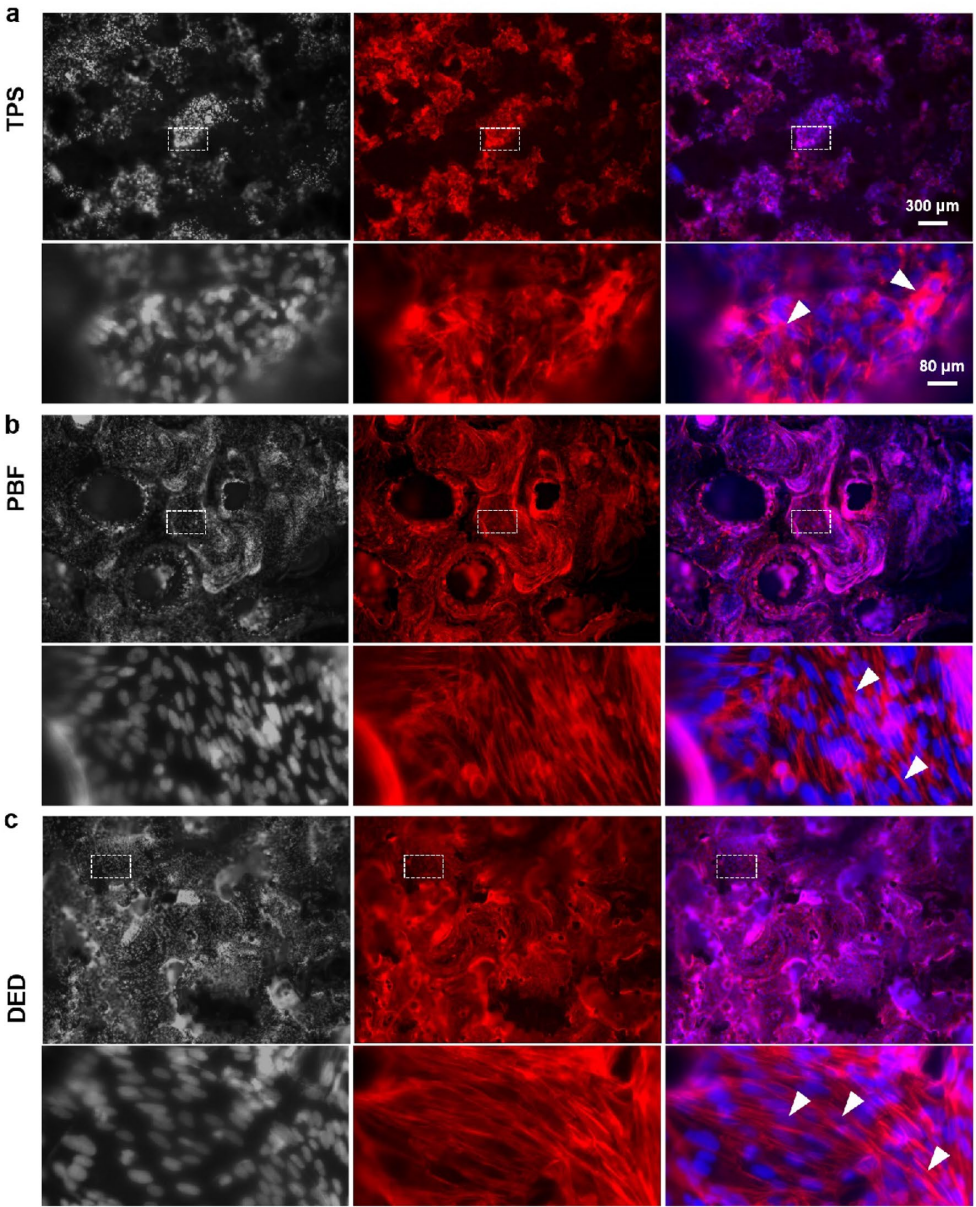
Immunofluorescence images of cells grown on (**a**) TPS, (**b**) PBF, and (**c**) DED surfaces. Nuclei are shown in gray (Hoechst 33324) and actin cytoskeletons in red (Rhodamin phalloidin). The nuclei are shown in blue in the merge channel. The cells were visualized using a wide-field fluorescence microscope (Leica TCS SP5).

Previous studies have demonstrated that the geometry of scaffold pores can influence cellular adhesion, proliferation, and differentiation^40^. Hence, we identified how the geometry of pores in TPS, PBF, and DED coatings affected cellular adhesion and proliferation. To investigate cellular adhesion properties, cells attached to the porous coating surface structures were fluorescently labeled and imaged. As shown in Fig. 4, actin stress fibers were labeled with phalloidin (red) and nuclei were labeled with Hoechst 33342 (gray, and blue in the merged images). Cells appeared to adhere firmly onto the porous coating surface and spread along the surface topography (Fig. 4 and Supplementary Fig. S3). Given that TPS, PBF, and DED coatings are fabricated using different processes, it seems natural that these three porous coatings have their own unique patterns. The TPS surface exhibited valley-like and peak-like topography, and most of the cells seemingly clumped together in a valley-like structure rather than in a peak-like structure (marked with a red circle in Supplementary Fig. S3a) (Fig. 4a and Supplementary Fig. S3a). Actin stress fibers were oriented in an irregular direction and the nuclei of cells overlapped with each other (marked with white arrow heads in Fig. 4a). On the other hand, cells on the PBS and DED coatings appeared to spread well onto Ti surfaces, with distinct actin stress fibers (marked with white arrow heads) (Fig. 4b,c). In addition, as expected based on the CCK-8 results, cell density was high on the DED surface, and the cells appeared to cover the porous coating surface more uniformly (Supplementary Fig. S3c), whereas the PBF surface had some patches without cells (marked with red stars in Supplementary Fig. S3b).

Through the observation of surface topography and cellular morphology, we confirmed that the DED coating had a more favorable environment for cells to spread and proliferate, compared to the TPS or PBF coating. In particular, the high porosity of the DED coating and the resulting large surface area seem to make it easier to achieve successful osseointegration for a porous coating implant^41^.

### In vivo bone histomorphometry

**Figure 5:**
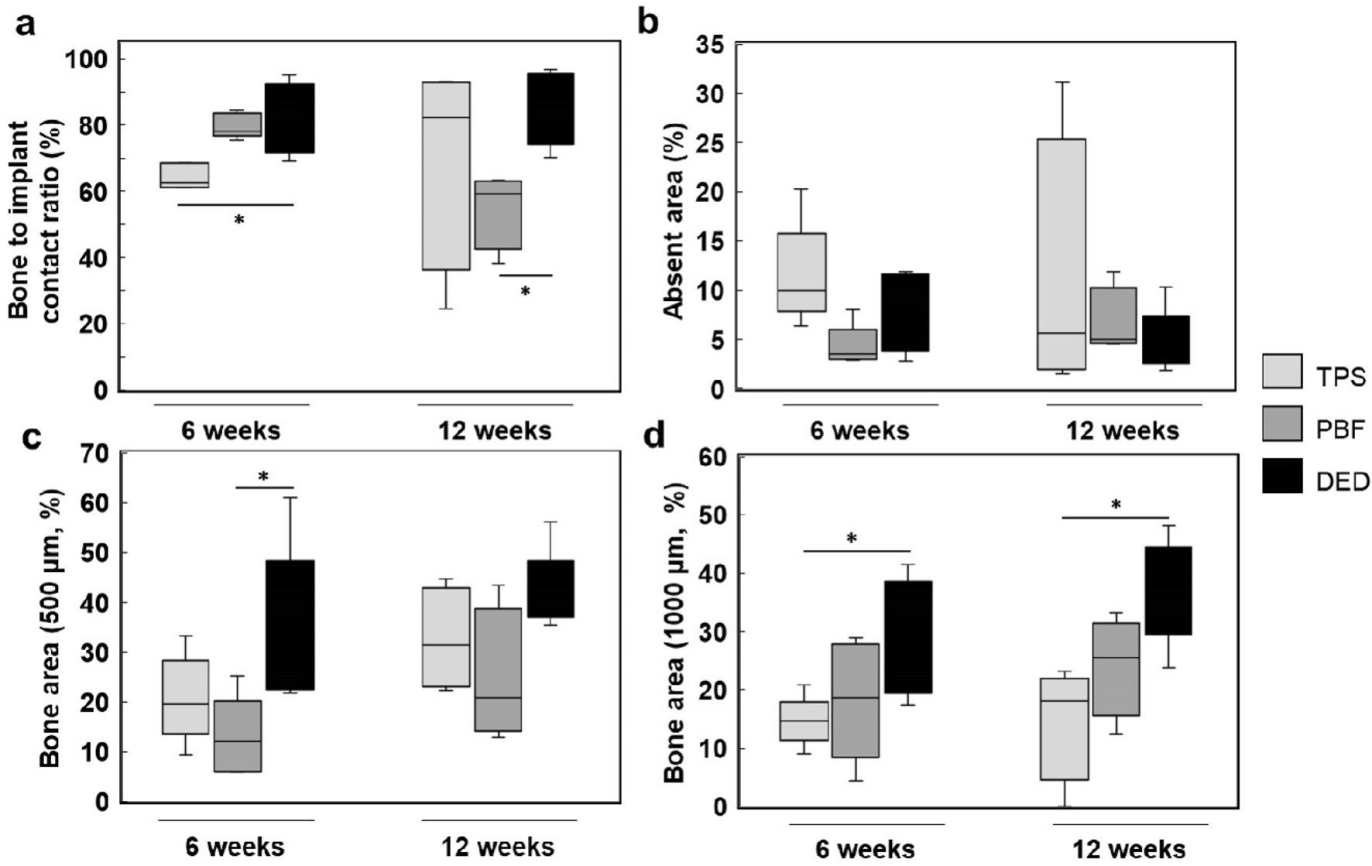
Results of bone histomorphometry. (**a**) Bone-to-implant contact (BIC); the DED group showed significantly higher BIC than the other groups 6 weeks after implantation, and higher BIC than the PBF group at 12 weeks. (**b**) Absent area; there was no significant difference across the three test groups at 6 and 12 weeks. (**c**) 500 µm bone area; the DED group showed higher osseointegration than the PBF group in 500-µm bone area at 6 weeks. (**d**) 1000 µm bone area; the DED group showed higher osseointegration than the TPS group at 6 and 12 weeks (*p < 0.05).

As in vitro studies showed that DED specimens exhibited the best outcome in terms of bone cell growth among the three porous coatings tested, we further compared the efficacy of osseointegration through in vivo studies. Interestingly, but perhaps not surprisingly, the DED group showed significantly higher bone-to-implant contact (BIC) than the other groups 6 weeks after implantation (DED: 81.31% ± 10.93%, PBF: 79.78% ± 3.70%, TPS: 64.35% ± 3.81%, *p* = 0.0092) (Fig. 5a). This difference in BIC between the coating types disappeared 12 weeks after implantation (Fig. 5a). There was no significant difference between the three different coating types with respect to the absent area, which can be related to osteolysis (Fig. 5b). The DED coating showed a larger newly formed bone area than the PBF coating within the 500-µm zone (Fig. 5c), and also exhibited a larger area within the 1000-µm zone compared with the TPS coating (Fig. 5d). The tendency of DED coatings to have a larger bone area compared to other coating types remained similar even when the comparison area was extended to the 2000-µm zone (Supplementary Fig. S4). Although the statistical significance varied according to the comparison group, the data confirmed that the DED coating showed higher osseointegration than the TPS and PBF coatings.

In addition, we checked whether the high efficiency seen in DED coatings was consistently maintained throughout the early and late osseointegration. Therefore, two different time points (6-week and 12-week time points) were selected and observed for histological study, and no significant difference was observed in the trend between the two-time points. As a result, we concluded that DED coating showed consistently good osseointegration performance throughout the early and late periods of osseointegration. As such, all three specimens appear to enable fairly stable osseointegration, the overall data show that the DED coating is more beneficial for bone ingrowth. To determine the actual bone ingrowth efficacy, further experiments should be conducted, using more meticulously designed implants, on larger animals such as Beagle dogs.

## Conclusions

Since various porous coating technologies for orthopedic implants have been developed, it is becoming increasingly important to evaluate the efficacy of new coating methods. We performed a systematic mechanical and biological comparative study on the DED method and other commercially available technologies, i.e., TPS and PBF. Although DED-coated specimens seem to show lower fatigue life compared to TPS-coated specimens, in terms of coating stability, DED coatings appear to have high bonding stability. Given the fact that this fatigue cycle of DED can be improved using the HIP process, we believe that DED can be produce coatings with sufficiently high fatigue strength for cementless orthopedic implants in the clinic. In addition, DED coatings show higher efficiency in osseointegration both in vivo and in vitro, compared with coatings made using other methods; this appears to be due to the more favorable surface topography (or pore morphology) for cells to spread and proliferate, and the high porosity to allow easier bone ingrowth. In conclusion, the results of this study suggest that DED is a promising new porous coating method that can enhance osseointegration of cementless bioimplants. Given the significant increase in patient life expectancy over the past few decades, we expect DED-coated implants to be innovative alternatives to conventional coating methods.

## Materials and methods

### Specimen preparation

For comparison, we prepared three types of test coupons based on different manufacturing technologies (DED, PBF and TPS). Each test coupon was cylindrical in shape and consisted of a solid substrate and a porous coating surface layer. For in vitro tests, the solid substrates were made with a diameter of 14.6 mm and a height of 4.0 mm, and for in vivo tests, with a diameter of 6 mm and a height of 2.0 mm. The PBF porous coating surface layer was designed to have a 1.0 mm thickness, which was also the target for the DED and TPS layers (Supplementary Fig. S5).

To fabricate DED specimens, a porous surface layer was laminated on CoCr solid substrate using a DED metal 3D printer (MPC-mini, Insstek, Korea) with pure Ti powder (grade 2, ASTM F1580). The process parameters were set as follows: 70 W laser power, 1500 mm/min scan speed and 2.2 g/min powder feeding rate. To design the porous part of PBF specimens, we scanned an actual cancellous structure in the proximal tibia of a rabbit using a micro computed tomography (micro-CT) system (TVX-IMT225-RC-S2, Tech Valley, Republic of Korea) and employed commercial reconstruction software (Mimics 22.0 & 3-matic 14.0, Materialise, Belgium). Then, the specimen was fabricated using a PBF metal 3D printer (SLM280HL, SLM solution, Germany) with Ti-6Al-4V powder (grade 9, ASTM F136). The process parameters were as follows: 350 W laser power and 1400 mm/s scan speed. In the TPS, the porous layer was laminated on a CoCr solid substrate using a commercially available TPS coating with pure Ti powder (grade 2, ASTM F1580). The Ti powder was injected into the plasma gas stream generated with an electric arc gun with a temperature of about 20,000 °C. Then, the molten Ti particles impacted onto the CoCr substrate with high kinetic energy and were coated.

### Characterization of porous structure

Cross-sectional images of specimens were obtained via micro-CT scanning (SkyScan1173, Bruker, Belgium). The pore size of each porous surface coating type was measured by manually segmenting the pores from the cross-sectional images using ImageJ software (National Institutes of Health, USA). Then, based on these 2D cross-sectional images, we reconstructed 3D shapes to measure the porosity and layer thickness of the specimens using Mimics 22.0 and 3-matic 14.0 software (Materialise).

### Sample size calculation for in vitro and in vivo studies

For reliable in vitro and in vitro studies, we determined the minimum sample size using G*Power 3.1 software (Heinrich-Heine University, Dusseldorf, Germany), based on similar studies that we have performed previously^41–43^. The minimum sample size for each in vitro study was calculated (α = 0.05, β = 0.2) based on the mean and standard deviation of the OD values of the CCK-8 assay, and the minimum sample size for each in vivo study was determined based on the mean and standard deviation of BIC. For each in vitro study, six experiments were needed as the minimum requirement to ensure 80% power; hence, to meet the minimum requirement, every in vitro experiment was repeated approximately nine times. For each in vivo study, five experiments with each test group were needed as the minimum requirement to ensure 80% power. Therefore, five rabbits were randomly allocated to six different experimental conditions: DED 6 weeks, DED 12 weeks, PBF 6 weeks, PBF 12 weeks, TPS 6 weeks, and TPS 12 weeks.

### Fatigue cycle test

The fatigue cycle was measured via three-point bending tests using an Instron^®^ 8872 system (Instron, USA). The specimens were mounted to a jig attached to the Instron^®^ 8872 with the porous layer facing down. The fatigue tests were carried out at a frequency of 10 Hz and a stress ratio R of 0.1 (minimum stress 103.33 MPa and maximum stress 1033.33 MPa).

### Cell preparation for in vitro study

The human osteoblast cell line, Saos-2, was purchased from the Korean Cell Line Bank (KCBL). Saos-2 cells were cultured in Minimum Essential Medium (MEM, Welgen) supplemented with 10% fetal bovine serum (FBS, #S001-01, Welgen, Republic of Korea) and 1% anti-anti (Gibco, Thermo Fisher Scientific, Inc., Waltham, MA, USA).

DED, PBF, and TPS test coupons were prepared and cleaned in six steps for in vitro experiments. Firstly, the coupons were sonicated twice in a solution with 1% Solujet (Alconox) at 45 °C. Then, the mixture was sonicated in distilled water twice at 45 °C. After sonication, the coupons were autoclaved at 120 °C for 30 min and dried in a dry oven at 60 °C. Then, each experimental coupon was placed in a 24-well plate for cell culturing. To minimize the number of cells that adhered to the surface of the plastic wells instead of the surface of the coupons, a non-surface-treated 24-well plate (#32024, SPL Life Science) was used and the coupons were designed to fit tightly into the 24-well plate hole. For the positive control, cells were cultured directly on a surface-treated 24-well plate (#30024, SPL Life Science). Saos-2 cells were seeded at a density of 2.4 × 10^4^ cells/well and incubated in a CO_2_ incubator at 37 °C.

### Cell proliferation and alkaline phosphatase (ALP) activity assays

Cell proliferation rates were assessed using CCK-8 (Dojindo Molecular Technologies, USA). Cell proliferation was tested at 3, 5, 7, 10, or 14 days of culture. At each time point, CCK-8 reagent (50 μL) was added to each well of 24-well plates, and the plates were incubated in a CO_2_ incubator at 37 °C for 90 min. The OD value was measured using microplate reader (BioTek™ Eon™ Microplate Spectrophotometers, USA) at 450 nm.

An ALP assay kit was purchased from Abcam (UK). After 5 or 14 days of cell culturing, cells were lysed twice with ALP assay buffer on ice for 10 min. The lysate was collected and centrifuged at 3000 rpm and 4 °C for 15 min. Then, the ALP assay kit was used, in accordance with the kit manual. The lysate was injected into a 96-well plate and 5 mM p-Nitrophenyl Phosphate (pNPP) solution was added. The Lysate-pNPP mixture was incubated at 25 °C for 60 min. The reaction was stopped by adding stop solution. The OD was measured using a microplate reader at 405 nm wavelength.

### Immunofluorescence imaging

After 14 days of culturing, cells were washed twice with phosphate-buffered saline (PBS), pH 7.4, fixed with 3.7% formaldehyde/PBS solution for 20 min at room temperature (RT), permeabilized with 0.2% Triton X-100 (Sigma, USA) for 15 min, and washed twice with PBS. To reduce non-specific binding, 5% normal goat serum/PBS was added to the sample, which was then incubated for 1 h at RT^44,45^. Rhodamine phalloidin (Invitrogen, Grand Island, NY, USA) in 5% normal goat serum/PBS was used to label actin stress fibers, and 5 μg/mL Hoechst 33342 (Sigma, USA)/PBS solution were used to label nucleus^46^. Since an inverted confocal microscope was used, we dropped the mounting solution (Vectashield^®^ Vector Labs., USA) and placed the coupons upside down on the cover glass bottom dish (SPL Life Science) to ensure that the cell-attached-surface was facing downward. The cells were visualized using a wide-field fluorescence microscope (DMi8, Leica, Germany) and a confocal microscope (TCS SP5, Leica, Germany). Using confocal microscopy, we detected the Hoechst-33342-stained nuclei with 450 nm emission/400 nm excitation wavelengths, and the topography of the specimen surface with the laser light reflected (580 nm emission/580 excitation) from the surface.

### Quantification of cell adhesion at initial stage

To compare the properties of different types of porous surfaces at the initial stage of cell-surface adhesion, the cells attached to the specimens were quantified 6 h after seeding. Saos-2 cells were seeded on DED, TPS, PBF, and well plate (as positive control) surfaces at a density of 7.2 × 10^4^ cells/well, and incubated for 6 h. After incubation, the coupon surfaces and well plate surfaces were washed with PBS vigorously to remove the non-adherent cells. The coupons were then placed in a new 24-well plate, and each well was filled with the cell culture medium and CCK-8 reagent. All the specimens were incubated with CCK-8 reagent for 90 min in a CO_2_ incubator at 37 °C, and the OD values of the media were measured using a microplate reader at 450 nm.

### Animal preparation

Thirty-six-week-old female New Zealand white rabbits with an average weight of approximately 4 kg were used in this study. The rabbits were individually housed in a temperature- (23 ± 2 °C), humidity- (60% ± 10%), and light-controlled environment, and provided with food and water ad libitum under a 12 h light cycle^41^. Animal care, surgical procedures, and all experimental protocols with in vivo animal model were performed in accordance with the National Institutes of Health guide for the care and use of laboratory animals. The study protocol was approved by the Ethics Committee on Animal experimentation at Samsung Medical Center (SMC 2018-0713-002). Also, this study was carried out in compliance with the ARRIVE guidelines. In total, thirty experiment animals were used for six different experiments: 6 weeks of DED (n = 5), TPS (n = 5), PBF (n = 5) and 12 weeks of DED (n = 5), TPS (n = 5), PBF (n = 5).

### Surgical procedure

**Figure 6:**
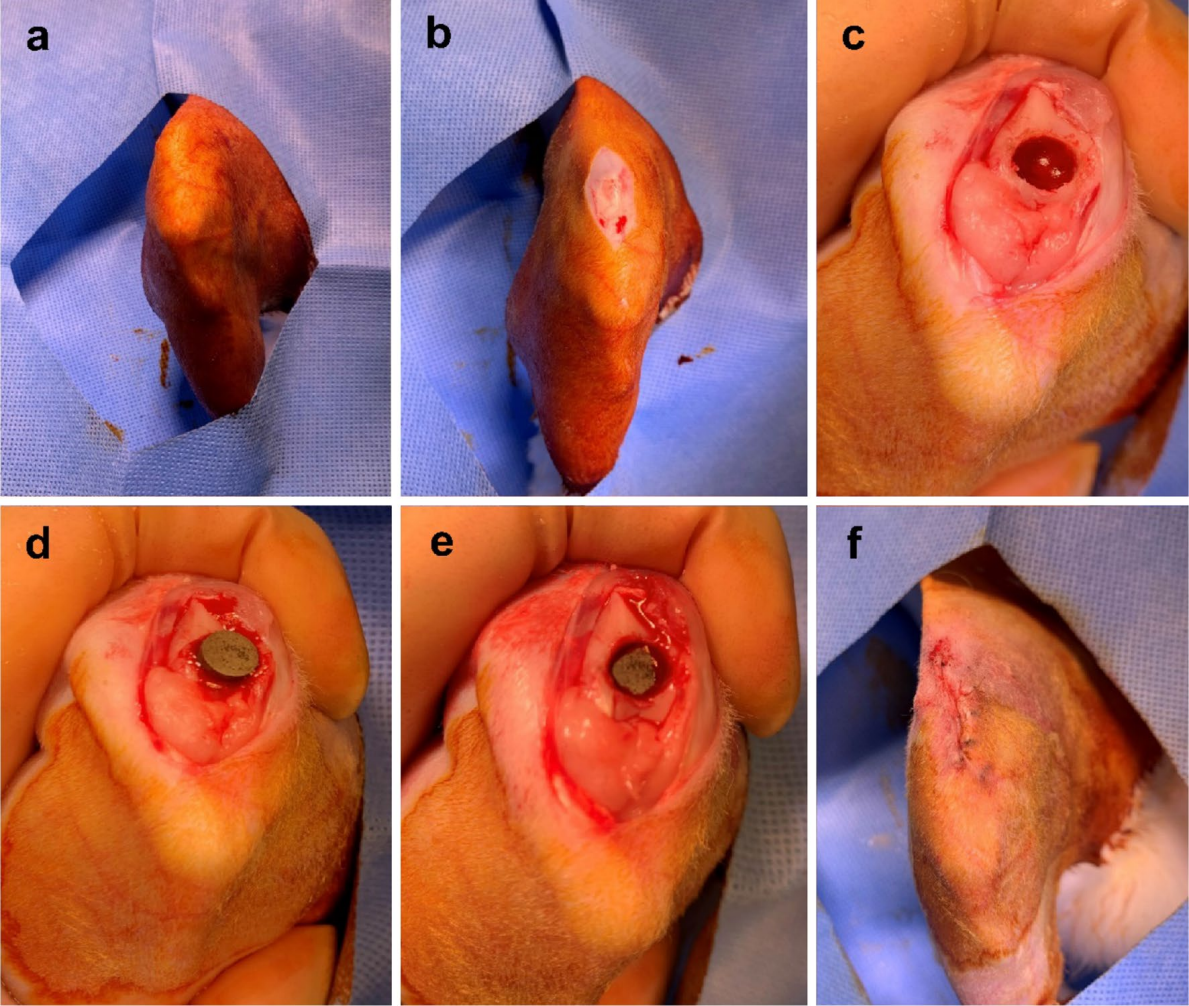
Surgical procedure for implanting specimens into rabbit trochlear grooves. (**a**) The right knee of each rabbit was shaved and sterilized with povidone–iodine. (**b**) In the supine position, the right legs were incised longitudinally from 2 cm above the knee joint to 1.5 cm below. (**c**) A hole with a 6 mm trephine burr was created on the proximal side of the trochlear groove. (**d**) A specimen was placed in the hole with the porous surface facing the cancellous bone. (**e**) The specimen was gently impacted to facilitate contact with the cancellous bone. (**f**) Patella reduction was performed and the incision was repaired.

General anesthesia was induced by intramuscular injection of ketamine (700 μL/kg) and xylazine hydrochloride (200 μL/kg). The right knee of each rabbit was shaved and sterilized with povidone-iodine (Fig. 6a). In the supine position, the right legs were incised longitudinally from 2 cm above the knee joint to 1.5 cm below (Fig. 6b).

On the superomedial side of the patella, the vastus medialis muscle was incised through the medial side patella and patella tendon to the proximal end of the tibial tuberosity. This exposed the trochlear groove and the condyle of the femur, with the patella sliding to the lateral side. A hole in the proximal side of the trochlear groove was created with a 6 mm trephine burr, taking care to ensure that the hole was gently reamed (Fig. 6c). Normal saline was sprayed during the reaming procedure to prevent thermal injuries around the bone and soft tissue. An experimental specimen was placed in the hole of the trochlear groove with the porous surface facing the cancellous bone (Fig. 6d). We gently impacted the specimen to facilitate contact with the cancellous bone (Fig. 6e). After implantation, patella reduction was performed. After checking the reduction status and knee motion, we repaired the joint capsule and subcutaneous tissue with Vicryl 2-0 (Fig. 6f). Finally, the wound was disinfected with povidone–iodine^41^.

### Postoperative care and sacrifice

After surgery, experimental rabbits were administered 0.6 mL/kg of cefazoline (Chongkundang, Seoul, Korea) and 1.8 mL/kg ketoprofen (UNIBIO tech, Seoul, Korea) intramuscularly thrice daily for 3 days. The rabbits were allowed to act freely within their cages after surgery. Subsequently, the rabbits were sacrificed after the planned number of weeks following implantation surgery. We injected ketamine (700 μL/kg) and xylazine hydrochloride (400 μL/kg) intramuscularly; this was followed by an intravenous injection of potassium chloride. Then, the right side of the distal femur was harvested, and the specimens were fixed in 10% neutral buffered formalin (Sigma-Aldrich Corp., St. Louis, MO, USA) for 2 weeks..

### Histologic slide manufacturing and staining

Specimens were cleaned with distilled water, and decalcification was performed using ethylenediaminetetraacetic acid (EDTA) solution (pH 9.0) (Zytomed systems GmbH, Berlin, Germany) for 5 weeks. After confirming the removal of calcium, the specimens were embedded in paraffin and sectioned to a thickness of 50 µm with a hard tissue slicer (Struers, Willich, Germany)^47^. The sections were stained with hematoxylin and eosin (Sigma-Aldrich) and Masson’s trichrome (Sigma-Aldrich) stain to visualize the contact surface and osseointegration. General specimen imaging and histomorphometric analyses were conducted at 40 × magnification (Supplementary Fig. S6)^41^.

### Bone histomorphometry

Light microscopy images were obtained using 12.5 × and 100 × objective lenses (BX 51, Olympus, Tokyo, Japan). The images were captured using a digital camera (CC-12, Soft Imaging System GmbH, Munster, Germany) attached to the microscope^48^. Specimens from different implants were analyzed using (1) Bone to implant contact (BIC): the percentage of direct contact surface between mineralized bone and the Ti porous coating surface (Supplementary Fig. S7a); (2) absent area: the percentage of non-contact area within the total area in a 1000-µm region (Supplementary Fig. S7b); (3) bone area (500 µm): the percentage of new bone formation and neovascularization area within the total area in a 500-µm region; (4) bone area (1000 µm): the percentage of new bone formation and neovascularization area within the total area in a 1000-µm region; and (5) bone area (2000 µm): the percentage of new bone formation and neovascularization area within the total area in a 2000-µm region (Supplementary Fig. S7c)^47,49,50^.

### Statistical analysis

The Kruskal–Wallis test was used to compare differences between experimental groups. For comparison between each experimental group, multiple Mann–Whitney U tests were used and adjusted with the Benjamini–Hochberg procedure^51^. For analysis within each group from 6 to 12 weeks, the Wilcoxon signed rank sum test was used. All analyses were performed using SPSS^®^ 25.0 software (SPSS, Chicago, IL, USA). A *p* value < 0.05 was considered significant.

## Supplementary Information

Supplementary Information 1.

Supplementary Information 2.
