# Supplementary material for: Enhanced osseointegration through direct energy deposition porous coating for cementless orthopedic implant fixation
Source: Sci Rep. 2021 Nov 16;11:22317. doi: 10.1038/s41598-021-01739-9 (PMC8595809; doi:10.1038/s41598-021-01739-9)
Supplement: Supplementary file 2 — Supplementary Information 2. [file 41598_2021_1739_MOESM2_ESM.pdf]

Supplementary figures

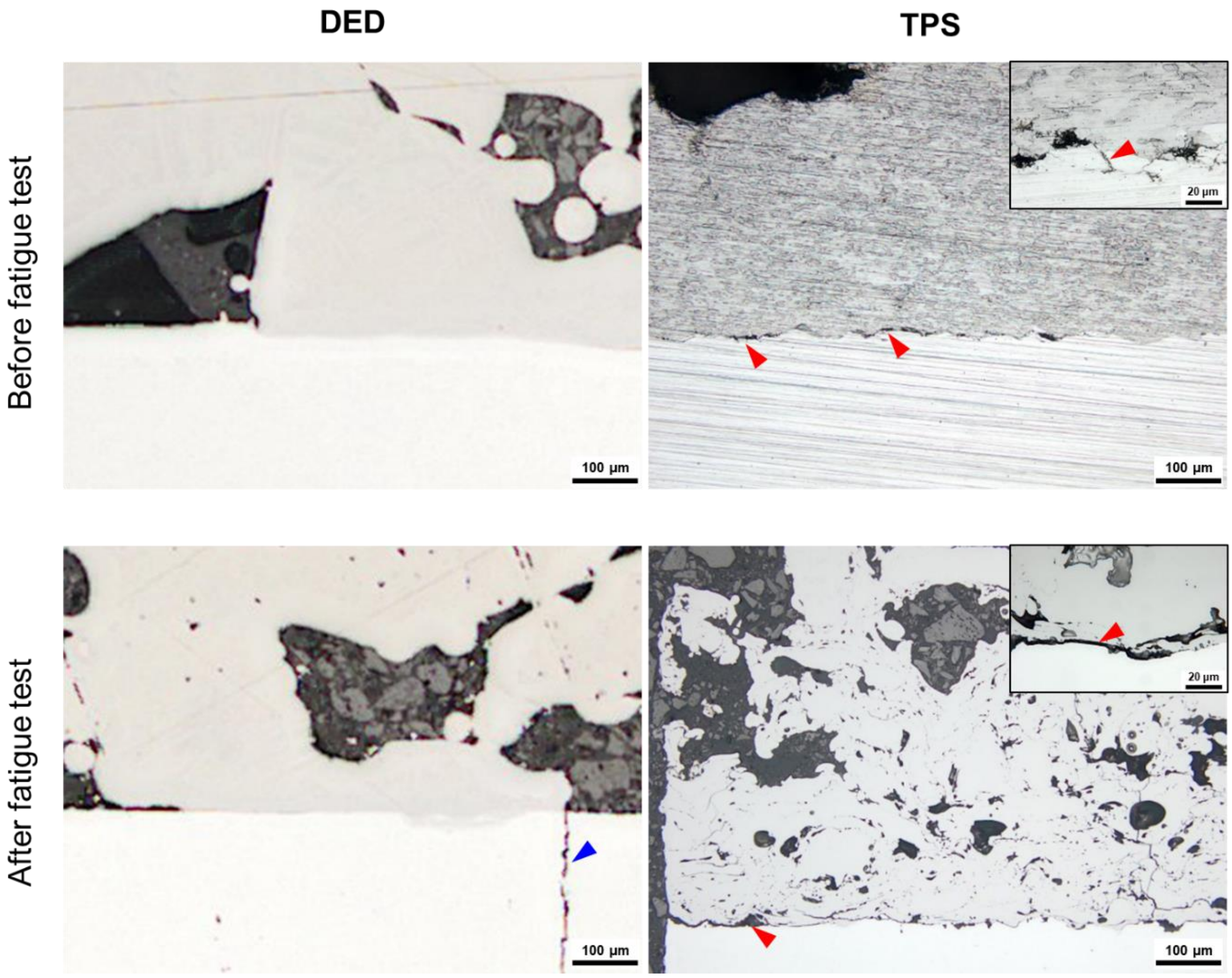

Figure S1. Cut cross-sectional images of DED and TPS before and after fatigue test. Red arrowhead indicates the site of debonding failure. Blue arrowhead indicates a crack caused by the fatigue test.

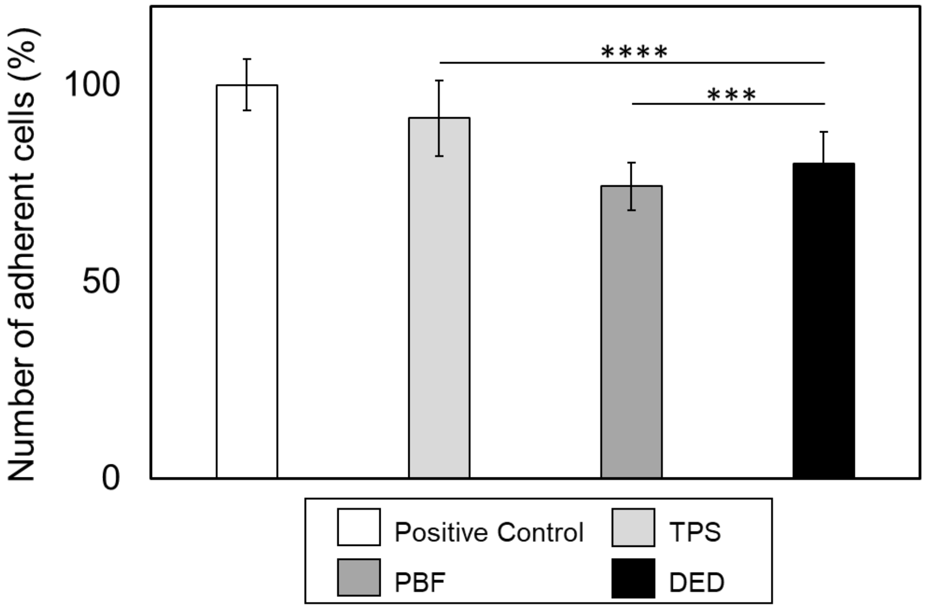

Figure. S2. Cell adhesion properties in the early stage on the different surface. Cell number was measured six hours after seeding. By CCK 8. Cell number are normalized by positive control and data are reported as mean ± SD (***p < 0.01. ****p < 0.0001).

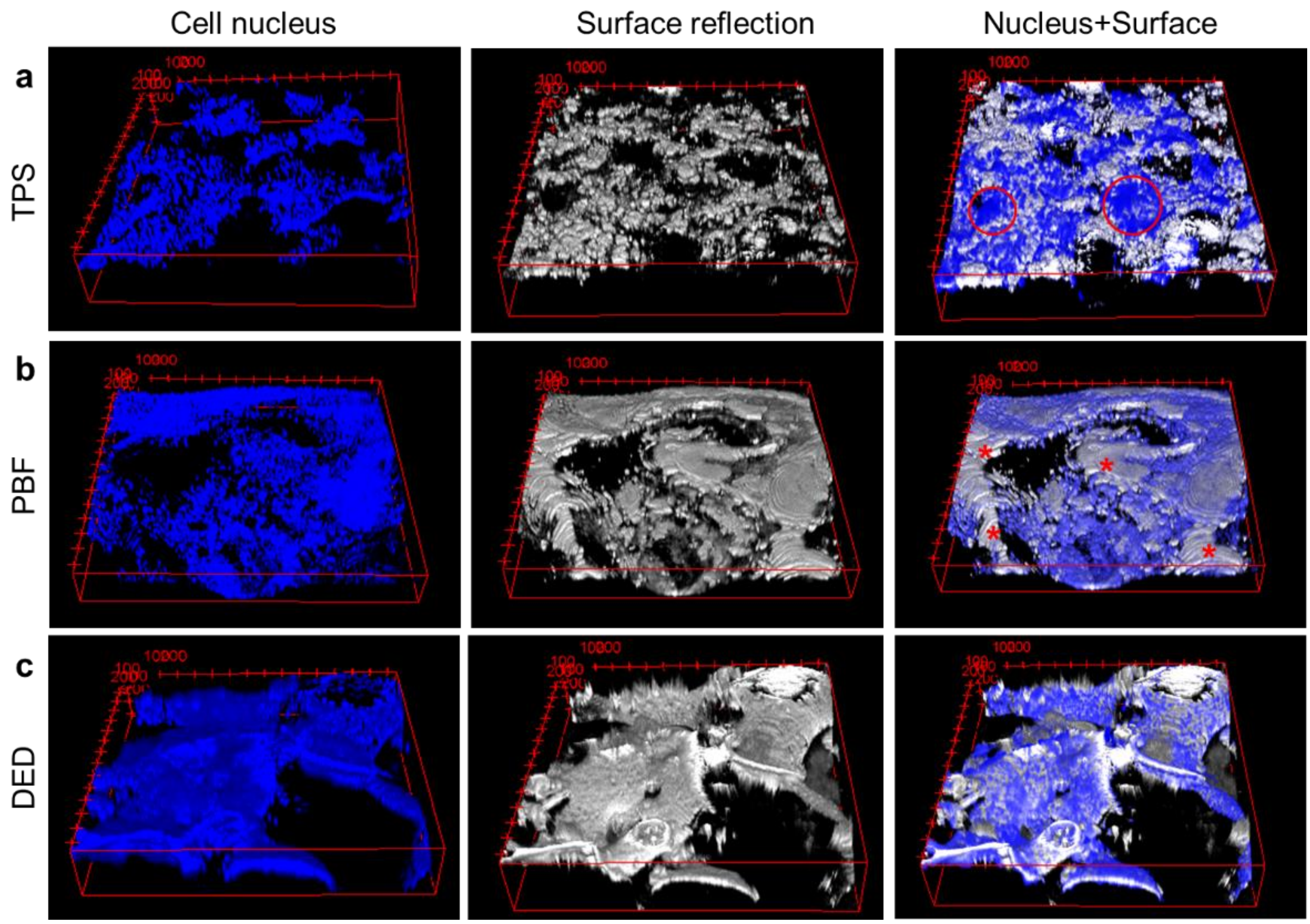

Figure. S3. Confocal images of cells grown on (a) TPS, (b) PBF and (c) DED surface. the nucleus is shown in blue (Hoechst) and the metal surface is shown in grey. The cells were visualized using a confocal microscope (Leica TCS SP5) Red circles indicate cell patches adhere on TPS surface. Red stars indicate patches without cells.

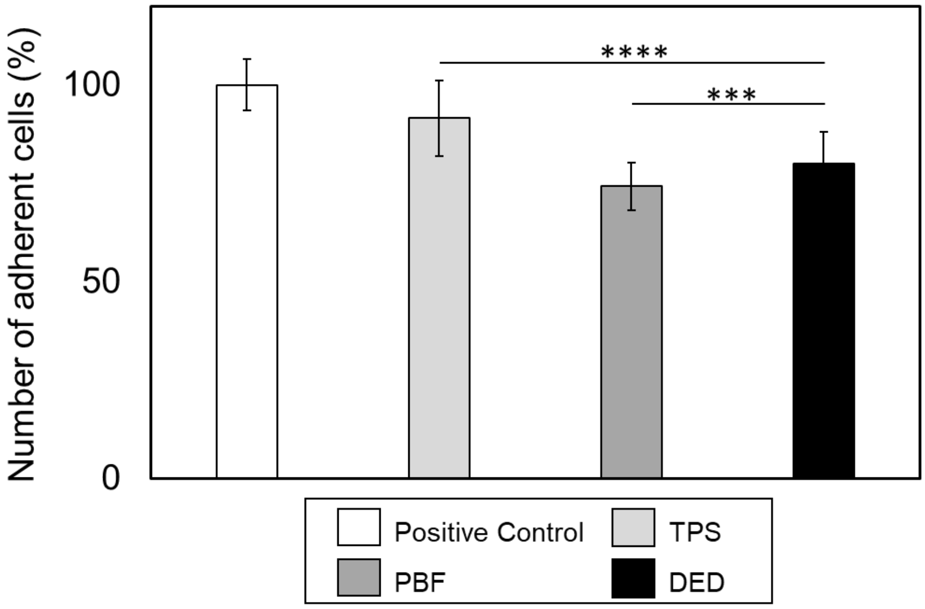

Figure. S4. Cell adhesion properties in the early stage on the different surface. Cell number was measured six hours after seeding. By CCK 8. Cell number are normalized by positive control and data are reported as mean ± SD (***p < 0.01. ****p < 0.0001).

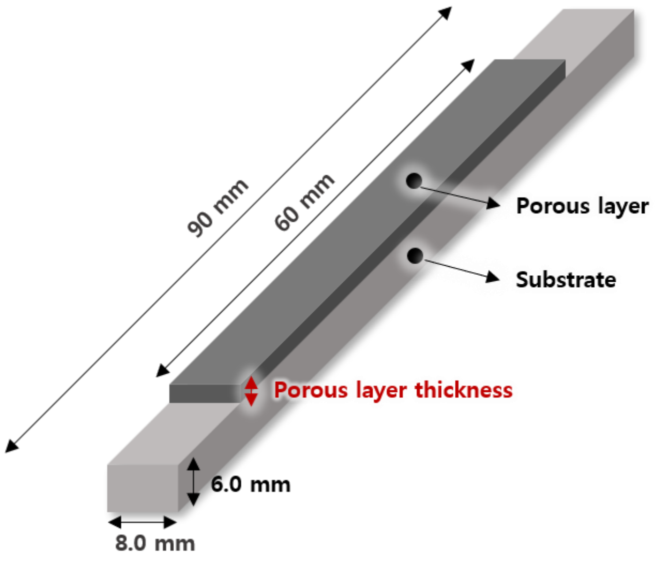

[DED specimen]

1. Materials:
   - Porous layer: Pure titanium
   - Substrate: CoCrMo F1537
2. Porous layer thickness: 1.0 mm

[TPS specimen]

1. Materials:
   - Porous layer: Pure titanium
   - Substrate: CoCrMo F1537
2. Porous layer thickness: 0.8 mm

[PBF specimen]

1. Materials:
   - Porous layer: Ti64 ELI
   - Substrate: Ti64 ELI
2. Porous layer thickness: 1.0 mm

Figure S5. Fatigue specimen size and dimensions for DED, TPS, and PBF.

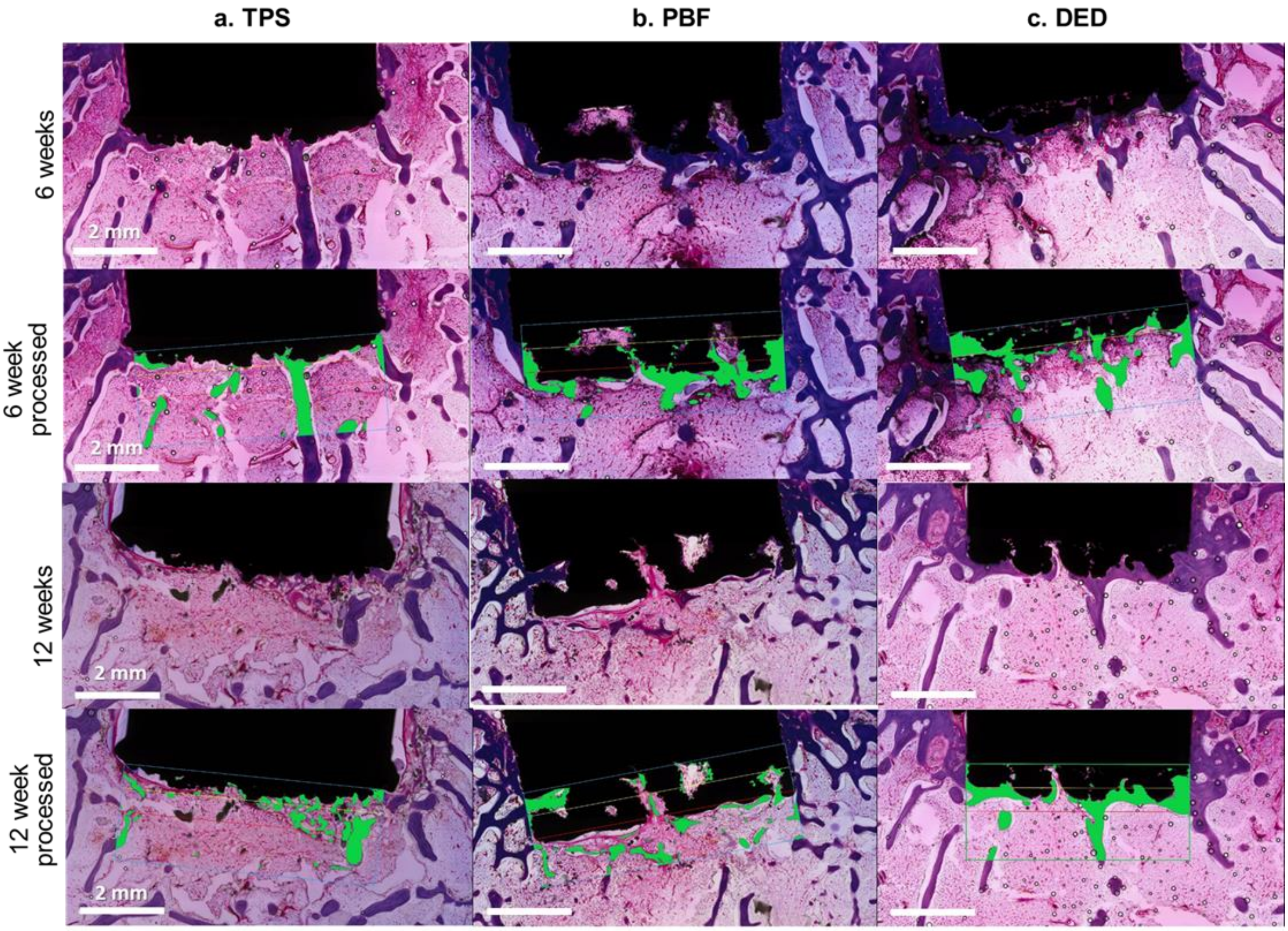

Figure S6 The representative Mason-trichrome staining mages of this study. a. TPS; b. PBF; c. DED. Green pixels indicate bone area.

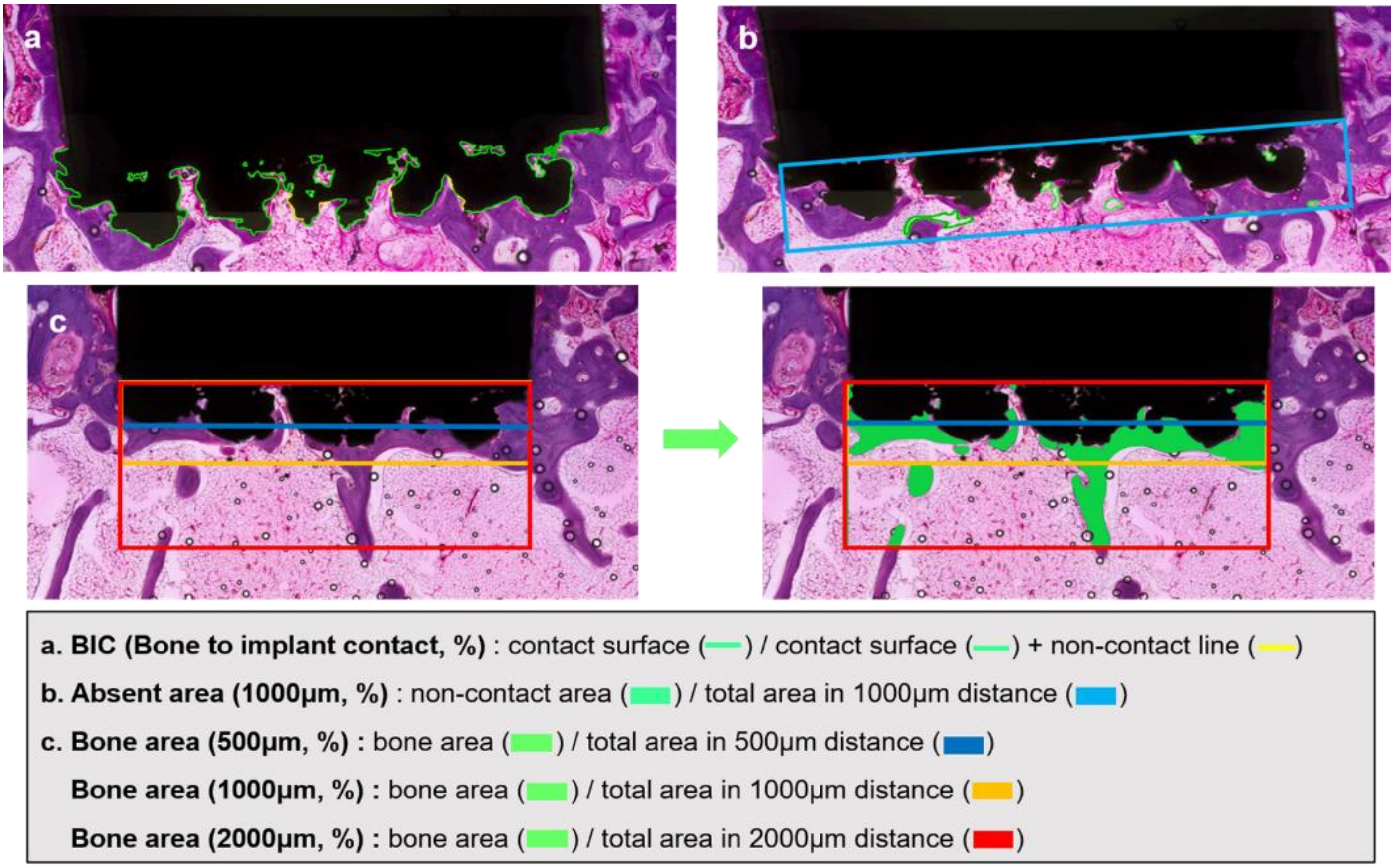

Figure S7 The process of bone histomorphometry. a. Bone-to-implant contact (BIC): determination of the percentage of direct contact surface between the mineralized bone and the porous surface; **b. Absent area**: determination of the percentage of non-contact area between the total area in a 1000 μm region; **c. Bone area** (1) bone area (**500-μm zone**): determination of the percentage of new bone formation and neovascularization area between the total area in a 500 μm region; (2) bone area (**1000-μm zone**): determination of the percentage of new bone formation and neovascularization area between the total area in a 1000 μm region; (3) bone area (**2000-μm zone**): determination of the percentage of new bone formation and neovascularization area between the total area in a 2000 μm region.
